# Acne

**Published:** 1886-01

**Authors:** 


ACNE, ITS ETIOLOGY, PATHOLOGY AND TREATMENT, A PRACTICAL TREATISE BASED ON THE STUDY OF ONE THOUSAND FIVE HUNDRED CASES OF SEBACEOUS DISEASE. *By* L. DUNCAN BULKLEY, A. M., M. D., *Physician to the New York Skin and Cancer Hospital, etc., New York and London*; G. P. PUTNAM'S SONS, 1885, PP. 280.

The author of this treatise has already made himself known to the profession at large by several useful contributions to dermatological literature, and, some years ago, by a translation of Neumann's hand-book of skin diseases, which has long been superseded by more modern treatises of wider scope. The author has here selected the relatively small field of disorders of the sebaceous glands, represented by acne as a type, for the field of his labors. The consequence is, that we find here a volume of over 200 pages, treating fully and well-nigh exhaustively of the topics practically and concisely discussed in a score of pages in most of the standard text-books of dermatology. Efforts of this kind, are, however, not without benefit to some. There is first the author, who is better for such full study of a particular field, and the embodying of the fruits of his own and of others' research in his pages. There is next the collector of dermatological literature of all sorts, who is pleased to be able to place a good monograph on acne upon his reference-shelves ; and there is last, the general practitioner, who, not content with what he finds more concisely given by others, and eager to prepare himself for a speeial case, invests with that purpose in view. But there is also a class of people actually harmed by books of this sort. It is they who, themselves suffering from the diseases discussed by any author, purchase his work in the hope to find in this way relief from their trouble. It is those who pay the largest price for their ignorance. There is no class of patients so hopelessly abandoned to the hypochondriacal and valetudinarian side of every disorder, as those who study their own diseases. The last and lowest stage of these unfortunates, is when they study medicine with the same end in view. Nowhere is there such a field for the growth of these morbid impulses, as in the class of disorders represented by acne, a class almost never associated with grave physical disease, but having a chief importance in relation merely to comeliness of the person.

The chapter here given on anatomy, and that also on the nosology of the disorders of the sebaceous glands, are particularly well written, and exhibit the fruits of some commendable pathological work. In the chapter, however, on etiology, the author ignores altogether the causation by ingested medicaments of the disease he discusses. Under the title of "Acne Artificialis," however, under which term are included the eruptions induced by both ingested and externally applied drugs, in another part of his book, he seems to put such confidence in the results of the studies of Pellizari, Thin, Duckworth, and others, as to justify him in setting the medicamentous rashes aside in a separate category by themselves. And yet it is certain that very few experts of any experience in this field will deny that nearly one half of all cases of acne, which come under their observation, have been aggravated by medicaments swallowed, purchased knowingly of druggists, or incorporated in many of the so-called blood, liver, and kidney-purifiers. Not only the iodine and bromine salts, but also arsenic and other medicinal substances figure in many cases as efficient producers of the disease. It is surprising that the author should consent to reduce this vast subject to the compass of a few paragraphs, seeing that a book fully as large as his might be well and instructively written on this practical point alone. But the great and growing field of preventive medicine calls for more devoted, more self-sacrificing, more unselfishly educated disciples than the average bookmaker. It is more lucrative to "treat," than to show how not to "treat." It pays better to have the reputation of "curing," than to watch with confidence and content the final involution of a disorder, whose satisfactory ending has been foretold by science!

With this explanation of the main defects of the book before us, it is just to say that, as regards most other points, the author has given a full and satisfactory review of the literature of his subject.

The formulary appended will prove useful to those who see many cases of disorders of the sebaceous glands; for those who see but few, the chapters on the management of the disease in general, may be consulted with profit. The typographical appearance of the work is all that could be desired.

